# Biological Characterization and DIVA Potential of Three Rough *Brucella melitensis* Vaccine Strains

**DOI:** 10.3390/vaccines13080857

**Published:** 2025-08-13

**Authors:** Jinyue Liu, Yi Yin, Xinmei Yang, Mengsi Li, Jing Qu, Shaohui Wang, Yanqing Bao, Jingjing Qi, Tonglei Wu, Mingxing Tian

**Affiliations:** 1 Hebei Key Laboratory of Preventive Veterinary Medicine, College of Animal Science and Technology, Hebei Normal University of Science and Technology, Qinhuangdao 066600, China; liujinyue0630@163.com; 2 Shanghai Veterinary Research Institute, Chinese Academy of Agricultural Sciences, Shanghai 200241, China; yinyisonia@126.com (Y.Y.); 15178100449@163.com (X.Y.); 15236611120@163.com (M.L.); ameliequ@163.com (J.Q.); shwang@shvri.ac.cn (S.W.); ybao@shvri.ac.cn (Y.B.); qijingjing@shvri.ac.cn (J.Q.)

**Keywords:** rough-type *Brucella*, virulence, vaccine, GntR, differential diagnosis

## Abstract

**Background:** Brucellosis is a zoonotic bacterial disease primarily controlled through quarantine, culling, and vaccination. Live attenuated vaccines remain the most effective countermeasure, yet their application is limited by residual virulence and diagnostic interference. This study developed three rough-type attenuated *Brucella melitensis* mutants (G7, G8, G16) and evaluated their potential as DIVA (Differentiating Infected from Vaccinated Animals) vaccine candidates. **Methods:** Rough phenotypes were characterized through heat agglutination, acridine orange staining, and immunoblotting. Macrophage cytotoxicity was assessed via LDH release assays, while RT-qPCR analyzed macrophage activation capacity. Mouse infection and immunization-challenge experiments, complemented by histopathology, evaluated residual virulence and protective immunity. Antibody profiles were determined by ELISA, and DIVA capability was verified using LPS-coated ELISA. **Results:** G7 and G8 exhibited complete rough phenotypes, whereas G16 retained partial O-antigen (semi-rough). All rough mutants induced macrophage cytotoxicity and activation. The strains showed attenuated virulence with no viable bacteria recovered from spleens at 4 weeks post-inoculation. Histopathology revealed no liver lesions at 6 weeks post-inoculation. Immunized mice predominantly produced IgG2a-dominated Th1-type responses. The immune protection levels of G7 and G16 matched the reference vaccine M5–90Δ26, while G8 showed slightly lower efficacy. LPS-ELISA effectively differentiated vaccinated from infected animals via concurrent IgM/IgG detection. **Conclusions:** This study demonstrates that the rough-type *B*. *melitensis* mutants G7 and G16 serve as promising DIVA vaccine candidates, offering strong protection with low residual virulence while enabling serological differentiation between vaccinated and infected animals, highlighting their potential as effective vaccines for brucellosis control.

## 1. Introduction

Brucellosis is a worldwide zoonotic bacterial disease affecting both humans and animals, with endemic persistence in regions such as Africa, Latin America, the Middle East, Asia, and the Mediterranean basin [1,2]. The disease imposes substantial economic burdens, particularly in developing countries, due to livestock reproductive failures such as abortion and infertility [3]. The causative agent, *Brucella*, is a Gram-negative, non-encapsulated coccobacillus [4]. Unlike many pathogenic bacteria, *Brucella* lacks classical virulence factors such as exotoxins, fimbriae, plasmids, and drug-resistant forms [5]. Instead, lipopolysaccharide (LPS) has emerged as a critical virulence determinant, influencing bacterial pathogenicity, biological phenotypes, and immune evasion [6]. Based on the integrity of the O-antigen structure in LPS, *Brucella* can be classified into smooth *Brucella* and rough *Brucella*. The rough *Brucella* can be further subdivided into complete rough *Brucella* and semi-rough *Brucella*. The complete rough *Brucella* lacks the entire O-antigen structure of LPS, whereas the semi-rough *Brucella* retains partial O-antigen structure. Notably, LPS contributes to the residual virulence of commercial *Brucella* vaccines and complicates serological differentiation between infected and vaccinated animals (DIVA) [7,8,9].

Live attenuated vaccines, including *B. abortus* S19, *B. melitensis* Rev.1, and *B. abortus* RB51, remain the cornerstone of brucellosis control programs [10]. However, their application faces significant challenges. Vaccines such as S19 and Rev.1 retain residual virulence, posing potential risks to human safety. Additionally, their smooth LPS phenotype elicits strong antibody responses, interfering with DIVA diagnostics. While the rough strain RB51—lacking O-antigen—serves as a DIVA-compatible vaccine, its rifampicin resistance limits therapeutic options in cases of accidental human exposure [10]. Furthermore, RB51, a *B. abortus* strain, is mainly used for immunizing cattle. *B*. *melitensis* primarily infects goats and sheep, yet no DIVA vaccine is currently available for controlling *B. melitensis* in small ruminants—underscoring the urgent need for novel vaccine development.

GntR family transcription regulators are widespread bacterial transcription factors. These proteins typically contain two functional domains: a conserved N-terminal DNA-binding domain with a helix-turn-helix (HTH) motif and a C-terminal effector-binding or oligomerization domain. In *Brucella*, several GntR regulators have been linked to bacterial virulence [11]. In this study, we serendipitously identified three LPS-deficient *Brucella* mutants (Δ*gntR7*, Δ*gntR8*, and Δ*gntR16*, designated G7, G8, and G16) while investigating GntR-family transcriptional regulators involved in virulence. We characterized their biological properties, residual virulence, and protective efficacy, evaluating their potential as DIVA vaccines. Our findings provide critical insights for the development of next-generation *Brucella* vaccines with improved safety and diagnostic compatibility.

## 2. Materials and Methods

### 2.1. Strains and Cell Lines

The *B. melitensis* parental strain M5 and vaccine strain M5–90Δ26 were obtained from the Chinese Veterinary Culture Collection Center (CVCC, Beijing, China). *Brucella* strains were cultured in tryptic soy agar (TSA) or broth (TSB) (Difco, Franklin Lakes, NJ, USA) at 37 °C under 5% CO_2_. All live *B. melitensis* experiments were performed in a biosafety level 2 (BSL−2) facility at SHVRI. *Escherichia coli* DH5α (TIANGEN, Beijing, China) was grown in Luria–Bertani (LB) medium. The murine macrophage cell line RAW264.7 (ATCC TIB−71, Manassas, VA, USA) was maintained in Dulbecco’s modified Eagle’s medium (DMEM; Gibco, Grand Island, NY, USA) supplemented with 10% (*v*/*v*) heat-inactivated fetal bovine serum (ExCell, Suzhou, China) at 37 °C in a 5% CO_2_ atmosphere. Strains and plasmids used in this study are listed in Table 1.

### 2.2. Primers and PCR Conditions

All primers used for PCR and RT-qPCR in this study are listed in Table 2. The PCR conditions were as follows: denaturation at 98 °C for 10 s, annealing at 55 °C for 15 s, and extension at 72 °C for 30 s. The qPCR conditions consisted of an initial denaturation at 95 °C for 30 s, followed by 40 cycles of denaturation at 95 °C for 10 s, and combined annealing/extension at 60 °C for 30 s.

### 2.3. Flow Diagram of Experiment

**Figure 1:**
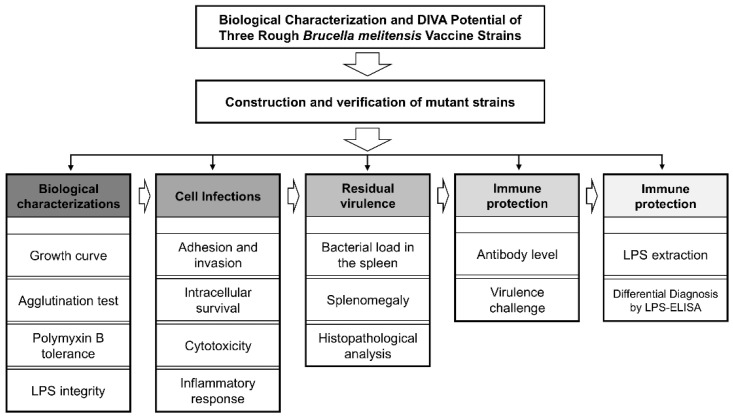
Flow chart of the experiment.

In this study, we constructed three rough-type *Brucella* strains (G7, G8, and G16) and subsequently evaluated their potential as vaccine candidates, following the experimental workflow outlined in Figure 1.

### 2.4. Construction of Gene Deletion Mutants

Gene deletion mutants (Δ*gntR7*, Δ*gntR8*, and Δ*gntR16*) were generated using a suicide plasmid-based approach as previously described [12]. Briefly, upstream and downstream fragments of each target gene (*gntR7*: BM28_RS10400; *gntR8*: BM28_RS08440; *gntR16*: BM28_RS14945) were amplified using primers GntR-UF/UR and GntR-DF/DR, respectively. These fragments were fused via overlap PCR (primers GntR-UF/DR) and cloned into the linearized pKB plasmid using the ClonExpress II One Step Cloning Kit (Vazyme, Nanjing, China). The resulting plasmid was electroporated into *B. melitensis* M5, with mutants selected through sequential antibiotic resistance (50 µg/mL kanamycin) and counterselection (5% sucrose). Successful deletions were confirmed by PCR. Additionally, the PCR-amplified target gene deletion fragment was gel-purified and subsequently sent to Tsingke Biotechnology Co., Ltd. (Beijing, China) for Sanger sequencing to confirm the deletion strain.

### 2.5. Agglutination Tests

The *Brucella* strains were cultured at 37 °C for 48 h, and the bacterial suspension was incubated at 80 °C for 2 h in a water bath to evaluate the agglutination. The heat-killed bacterial suspension was subjected to two washes with PBS, followed by a 10-fold concentration through centrifugation. A 50 µL bacterial suspension was mixed with a 50 µL 0.1% acridine yellow solution, and the mixture was incubated for five minutes to determine the autoagglutination [7,13].

### 2.6. Western Blotting

Bacterial cultures were pelleted by centrifugation and concentrated 10-fold in sterile water. The suspensions were lysed in 5 × SDS loading buffer (Beyotime, Suzhou, China) by boiling for 10 min. Approximately 0.5 μg of total proteins and LPS from the bacterial lysates were separated on 12.5% SDS-PAGE gels and transferred to nitrocellulose membranes following standard protocols [14]. Membranes were blocked with 5% skim milk in PBS for 1 h at room temperature, then washed three times with PBST (PBS containing 0.05% Tween−20). Primary antibody incubations were performed overnight at 4 °C using mouse anti-O-antigen monoclonal antibody (A76 12G12 F12; 1: 200 dilution) or rabbit anti-GroEL polyclonal antibody (prepared in our lab; 1: 2000 dilution). After PBST washes, membranes were incubated for 1 h at room temperature with HRP-conjugated goat anti-mouse IgG antibody (Thermo Fisher Scientific, Waltham, MA, USA. #31430; 1: 20,000 dilution) or goat anti-rabbit IgG antibody (Abcam, Cambridge, MA, USA. #ab6721; 1: 20,000 dilution). Signals were developed using LumiQ ECL substrate (Share-Bio, Shanghai, China) and captured using a Tanon 5200 Imaging System (Tanon, Shanghai, China) with a 1 s exposure.

### 2.7. Bacterial Growth Curves

Bacterial suspensions were adjusted to 0.1 of the OD_600_ value in TSB, and then they were cultured at 37 °C at 200 rpm and the OD_600_ value was measured every 6 h; the records were used to create the growth curve. Growth curve experiments were performed in triplicate, with one representative dataset (n = 4 technical replicates) shown.

### 2.8. Polymyxin B Sensitivity Assay

The assay was adapted from a previously described method [12]. Bacterial suspensions were standardized to 5 × 10^5^ CFU/mL in PBS and treated with polymyxin B (Sangon, Shanghai, China) at final concentrations of 0.5, 1.0, and 2.0 mg/mL. PBS-treated samples served as negative controls. After incubation at 37 °C with shaking (200 rpm) for 1 h, serial dilutions were plated on TSA to manual enumerate viable bacteria. The survival rate was calculated using the following equation: survival rate (%) = (CFU treated/CFU control) × 100. CFU treated represents the CFUs from polymyxin B-exposed samples and CFU control denotes those from PBS-treated controls. All assays were performed in triplicate.

### 2.9. Adhesion, Invasion, and Intracellular Survival Assay

To evaluate the adhesion, invasion, and intracellular survival of M5 and its mutant strains, the RAW264.7 murine macrophage cell line was seeded in 24-well plates (Nest, Wuxi, China) at 3 × 10^5^ cells/mL and infected with *Brucella* strains at a multiplicity of infection (MOI) of 100. Bacterial adhesion was promoted by centrifugation, followed by incubation at 37 °C with 5% CO_2_ for 1 h. After washing three times with PBS, adherent bacteria were quantified by lysing cells with 200 µL of 0.25% Triton X−100 and plating on TSA for CFU enumeration. The resulting CFU values represent the number of adherent *Brucella*.

To assess invasion, extracellular bacteria were eliminated by treating cells with DMEM containing gentamicin (100 µg/mL) for 1 h. The cells were then lysed, and invasive bacteria were quantified via CFU counts. The resulting CFU values represent the number of internalized *Brucella*.

For intracellular survival analysis, the remaining infected cells were maintained in DMEM supplemented with 1% FBS and gentamicin (20 µg/mL). At 1, 8, 24, and 48 h post-infection (p.i.), cells were washed, lysed, and plated on TSA to determine intracellular bacterial loads.

### 2.10. Macrophage Cytotoxicity Assay

The cytotoxicity of *Brucella* infection on RAW264.7 macrophages was assessed by measuring lactate dehydrogenase (LDH) release. Cells were cultured and infected as previously described [15]. At 24 and 48 h p.i., culture supernatants were collected, and LDH release was quantified using a commercial LDH Cytotoxicity Assay Kit (Beyotime, Suzhou, China) according to the manufacturer’s instructions. The negative control (spontaneous LDH release) was established using uninfected cells, whereas the positive control (maximum LDH release) was determined using lysed cells. LDH release calculation formula: 100% × (OD_490_ of infected cells − OD_490_ of uninfected cells)/(OD_490_ of lysed uninfected cells − OD_490_ of uninfected cells). All assays were performed in quadruplicate wells.

### 2.11. RNA Extraction and qPCR

RAW264.7 cells were infected with *Brucella* strains G7, G8, G16, and M5 as described above. At 24 h p.i., total RNA was extracted using the VeZol-Pure Total RNA Isolation Kit (Vazyme), followed by DNA removal with the TURBO DNA-free Kit (Ambion, Austin, TX, USA). Reverse transcription was performed using the PrimeScript™ RT Reagent Kit (Takara) to synthesize cDNA. qPCR was conducted on the QuantStudio 3 Real-Time PCR System (Thermo Fisher) using ChamQ Universal SYBR qPCR Master Mix (Vazyme). The β-actin gene was used as the internal reference, and relative gene expression was calculated using the 2^−ΔΔCt^ method. Three independent biological replicates were performed for each experiment.

### 2.12. Evaluation of Residual Virulence

Bacterial suspensions were prepared by adjusting the OD_600_ value in TSB and subsequently diluting to a concentration of 1 × 10^7^ CFU/mL. Groups of BALB/c mice (n = 5) were inoculated intraperitoneally with 0.1 mL (1 × 10^6^ CFU) of M5 and mutant strains; PBS was injected as a negative control. At 2, 4, and 6 weeks p.i., mice were anesthetized and underwent retro-orbital blood collection prior to euthanasia. Spleens were aseptically harvested and homogenized in 3 mL of sterile PBS containing 0.25% Triton X−100 (*v*/*v*) using a tissue homogenizer. Serial ten-fold dilutions of the homogenate were plated on TSA and incubated at 37 °C with 5% CO_2_ for 3−5 days before the enumeration of CFU.

### 2.13. Enzyme-Linked Immunosorbent Assay (ELISA)

The levels of relevant antibodies in serum were determined using mouse serum obtained from the above animal experiments. The procedure was as follows: First, 50 mL of M5 bacterial suspension was cultured in TSB, before being heat-inactivated at 80 °C for 2 h. The bacterial precipitate was collected by centrifugation, washed twice with PBS buffer, and lysed by sonication to obtain M5 whole-bacterial protein. Each well of the 96-well ELISA plate was coated with 25 μg of heat-killed and sonicated M5 protein in carbonate buffer (pH = 9.6), incubated overnight at 4 °C [7]. The mouse serum samples with 200-fold dilution obtained from the experiments were used as the primary antibody; HRP-conjugated goat anti-mouse IgG (Thermo Fisher; 1: 20,000 dilution), IgG1, IgG2a and IgM (Abcam; 1: 20,000 dilution) was used as the secondary antibody.

Additionally, ELISA was used to detect the antibodies against LPS. The LPS were extracted from the phenol phase using the hot phenol method as previously described [16]. Each well of the 96-well ELISA plate was coated with 0.1 μg LPS of M5 in PBS incubated overnight at room temperature. HRP-conjugated goat anti-mouse IgG and IgM were used as the secondary antibody. The experimental method was conducted as previously stated [7].

### 2.14. Virulence Challenge

Fifty 6–8-week-old mouse were divided into five groups, and immunized with 10^5^ CFU of M5–90Δ26, 10^8^ CFU of three rough mutant strains or PBS as control, respectively. At 30 days and 45 days post-vaccination (p.v.), five mice of each group were intraperitoneally injected with 10^5^ CFU of M5 and sacrificed 2 weeks post-challenge. Blood was collected from the orbital sinus, and the spleen was aseptically harvested and homogenized; viable bacteria were enumerated on TSA as previously described. In addition, the liver tissue samples were fixed by 4% paraformaldehyde in properly fixed condition for 2 days. The histopathological examination of liver from mice infection was completed by Wuhan Servicebio Biotechnology Co., Ltd. (Wuhan, China).

### 2.15. Statistical Analysis

Data were analyzed using GraphPad Prism 9.0 (GraphPad Software, San Diego, CA, USA). Student’s *t*-test was used for pairwise comparisons, while one-way or two-way ANOVA, followed by Dunnett’s multiple comparisons test, was applied for group analysis. A *p*-value < 0.05 was considered statistically significant.

## 3. Results

### 3.1. Three Spontaneous Rough-Type Mutants of B. melitensis Exhibit Attenuated Virulence

**Figure 2:**
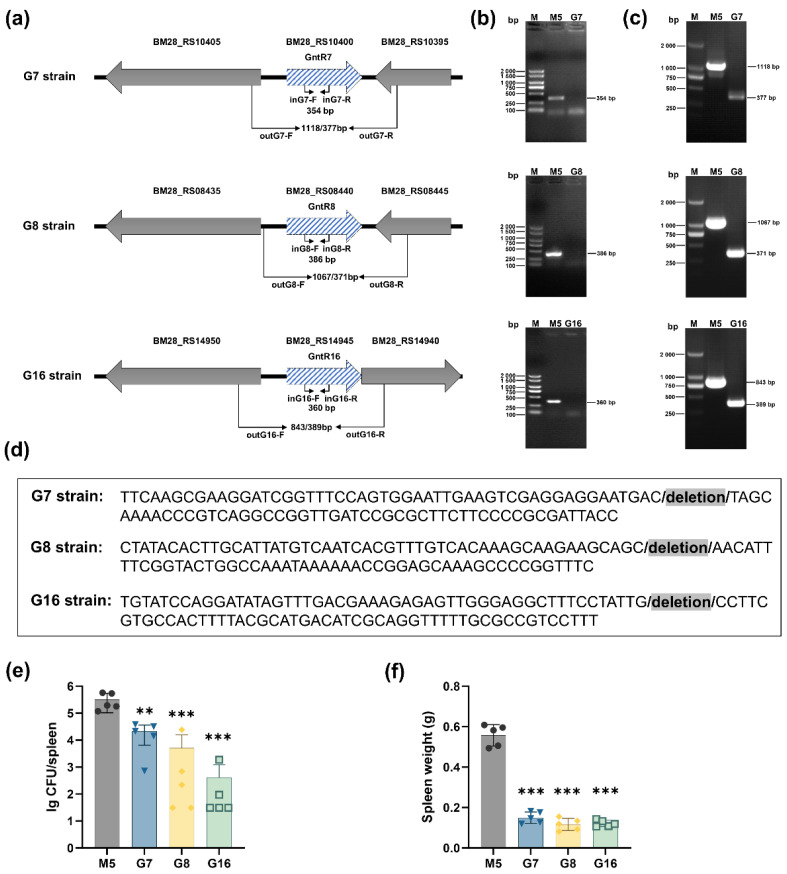
The construction strategy of G7, G8, and G16 strains and evaluation of their virulence. (**a**) Schematic of the construction strategy of G7, G8, and G16. (**b**) PCR identification of the G7, G8, and G16 strains using the inner primer pairs inG7-F/R, inG8-F/R, and inG16-F/R; (**c**) PCR identification of the G7, G8, and G16 strains using the outer primer pairs outG7-F/R, outG8-F/R, and outG16-F/R; (**d**) Sequencing analysis of the G7, G8, and G16 strains revealed their deletions in the target genes, as indicated by the gray-highlighted regions; (**e**) the bacterial load in the spleens of mice infected with G7, G8, and G16 for 2 weeks post-infection; (**f**) the spleen weight of the mice infected with G7, G8, and G16 at 2 weeks post-infection. Statistical significance was determined using one-way ANOVA (**, *p* < 0.01; ***, *p* < 0.001).

The genes of BM28_RS10400, BM28_RS08440, and BM28_RS14945 in *B. melitensis* strain M5 encode the GntR-family transcriptional regulators GntR7, GntR8, and GntR16, respectively (Figure 2a). Using bacterial homologous recombination, we successfully constructed three gene-deletion strains lacking *gntR7*, *gntR8*, and *gntR16*, designated as G7, G8, and G16 strains, respectively.

PCR verification was performed using inner and outer primer pairs (Figure 2a). The parental M5 strain (positive control) yielded PCR products of ~350 bp, ~380 bp, and ~360 bp for *gntR7*, *gntR8*, and *gntR16*, respectively, when amplified with inner primers, whereas no amplification was observed in the deletion strains (Figure 2b). With outer primers, M5 produced fragments of ~1100 bp, ~1000 bp, and ~840 bp for the respective genes, while the deletion strains yielded shorter products (~380 bp) (Figure 2c). Sequencing of these PCR products confirmed the successful deletion of the target genes, validating the construction of the G7, G8, and G16 mutant strains (Figure 2d).

Subsequently, we evaluated the virulence of the three mutant strains using a mouse infection model. Two weeks p.i., the bacterial load in the spleens of mice infected with the mutant strains was significantly lower than that in mice infected with the parental M5 strain (Figure 2e). Additionally, the spleen weights of mice infected with the mutant strains were markedly reduced compared to those infected with M5, suggesting that the mutants had a diminished ability to induce splenomegaly (Figure 2f). These data indicate that the virulence of the G7, G8, and G16 strains was significantly attenuated compared to the parental M5 strain. Unexpectedly, in situ complementation of the respective target genes in the mutant strains using a suicide plasmid failed to restore virulence, suggesting that the attenuation of G7, G8, and G16 may be due to other nonspecific factors.

**Figure 3:**
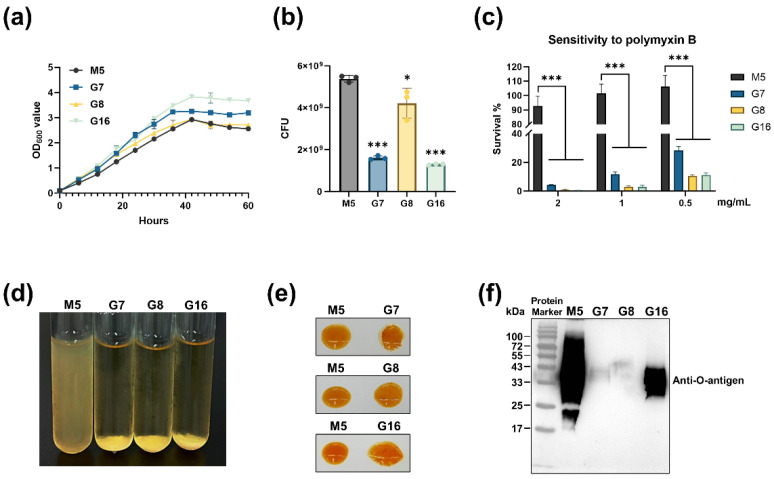
Analysis of the biological phenotypes of mutants. (**a**) Growth curves of G7, G8, G16, and M5 in TSB; (**b**) when the OD_600_ value was 1.0, the CFU of the mutant strains was determined; (**c**) sensitivity to polymyxin B; (**d**) heat agglutination test; (**e**) autoagglutination by acridine yellow staining; (**f**) Western Blotting detected the O-antigens of G7, G8, G16, and M5 strains. Statistical significance was determined using one-way ANOVA or two-way ANOVA (*, *p* < 0.05; ***, *p* < 0.001).

To assess the growth characteristics of the mutant strains, we measured their growth in TSB by monitoring the OD_600_ value. As shown in Figure 3a, the OD_600_ values of G7 and G16 were significantly higher than those of M5, while G8 exhibited a slight increase during the logarithmic phase. However, no obvious increase in turbidity was visually observed, leading us to hypothesize that changes in the outer membrane might affect the OD_600_ readings. To test this, we adjusted the OD_600_ value of the mutant strains to 1.0 and determined the CFU by plate-counting. As shown in Figure 3b, at an OD_600_ of 1.0, 1 mL of M5 suspension contained approximately 5 × 10^9^ CFU. In contrast, the CFU counts for G7, G8, and G16 were significantly lower, measuring approximately 1.6 × 10^9^, 4.2 × 10^9^, and 1.3 × 10^9^, respectively. To further investigate potential outer-membrane alterations, we assessed the susceptibility of the mutant strains to the cationic antimicrobial peptide polymyxin B. As shown in Figure 3c, G7, G8, and G16 exhibited significantly increased sensitivity to polymyxin B compared to M5. These findings suggest substantial structural changes in the outer membrane of the mutant strains.

Previous studies have reported that the O-antigen structure of *Brucella* LPS is unstable, and its loss can lead to a transition from a smooth (S-type) to a rough (R-type) phenotype, which affects bacterial absorbance and polymyxin B sensitivity [17,18]. Based on these observations, we hypothesized that G7, G8, and G16 might have undergone an S-to-R transition. To confirm this phenotypic change, we performed heat agglutination and acridine orange-staining assays. As shown in Figure 3d,e, the mutant strains exhibited pronounced heat-induced agglutination and autoagglutination by acridine orange staining compared to M5. To further verify the integrity of the O-antigen, we analyzed its structure by Western Blotting. As shown in Figure 3f, G7 and G8 completely lost their O-antigen, while G16 retained a partial O-antigen structure (hereafter, G7, G8, and G16 will be collectively referred to as rough *Brucella* strains.). Together, these results demonstrate that G7, G8, and G16 are three randomly isolated O-antigen-deficient attenuated *Brucella* strains.

### 3.2. Rough Brucella Strains Show Enhanced Adhesion and Invasion but Impaired Intracellular Survival

**Figure 4:**
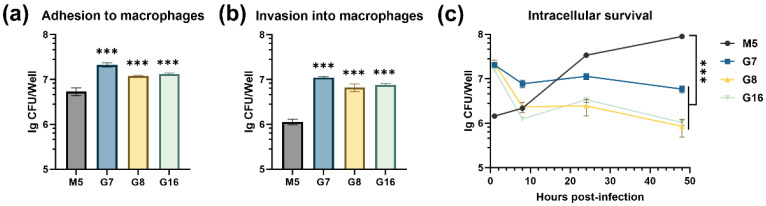
The ability of G7, G8, G16, and M5 to adhere, invade, and survive within macrophages. (**a**) Adhesion to RAW264.7 cells; (**b**) invasion into RAW264.7 cells; (**c**) intracellular survival within RAW264.7 cells. Statistical significance was determined using one-way ANOVA or two-way ANOVA (***, *p* < 0.001).

To evaluate the infectivity of rough *Brucella* strains in host cells, this study first assessed the adhesion and invasion abilities of three rough *Brucella* strains against murine macrophage RAW264.7 cells. The results showed that the number of viable bacterial CFUs recovered after adhesion to RAW264.7 cells was significantly higher in the three rough strains compared to the smooth strain M5 (Figure 4a). Similarly, following gentamicin treatment to eliminate extracellular non-adherent bacteria, the rough strains exhibited significantly higher viable intracellular bacterial counts (approximately 1 log10 CFU) compared to the M5 invasion group (Figure 4b). These findings indicate that the three rough *Brucella* strains significantly enhanced their adhesion and invasion abilities in RAW264.7 cells. Subsequently, this study further evaluated the intracellular survival ability of the rough *Brucella* strains. The results revealed a significant decline in intracellular survival for all three rough strains compared to the parental strain M5 (Figure 4c). Thus, the three rough *Brucella* strains exhibited weakened intracellular survival capacity.

### 3.3. Rough Brucella Strains Induce Macrophage Death and Significantly Activate Inflammatory Responses

**Figure 5:**
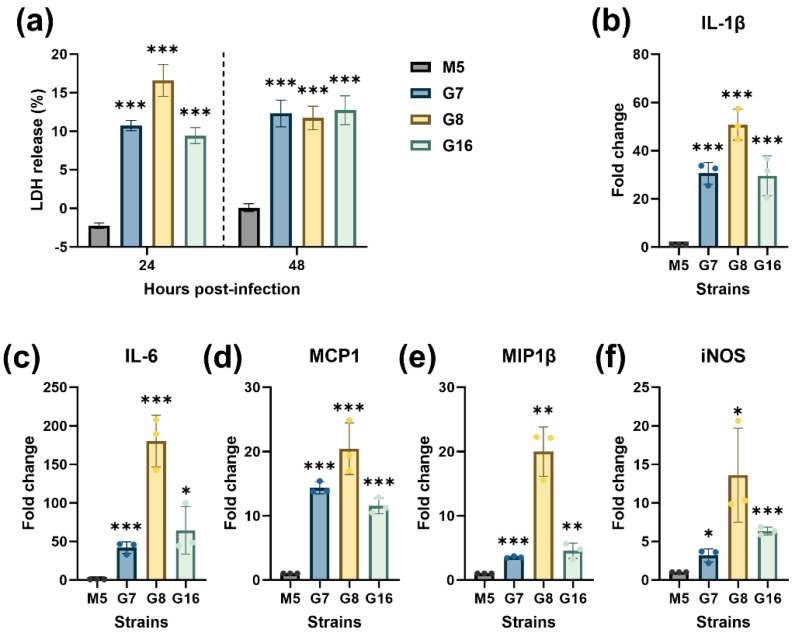
Rough-type *Brucella* induces macrophage death and activation. (**a**) LDH release from RAW264.7 cells infected with G7, G8, G16, and M5 strains at 24 and 48 h post-infection; (**b**–**f**) qPCR analysis of cytokines and chemokines expression in RAW264.7 cells infected with mutant strains and M5 at 24 h post-infection. Statistical significance was determined using one-way ANOVA or two-way ANOVA (*, *p* < 0.05; **, *p* < 0.01; ***, *p* < 0.001).

It has been reported that rough *Brucella* strains often cause macrophage death upon infection [15,19]. In this study, we evaluated the ability of three rough *Brucella* strains to induce macrophage death. LDH release assays showed that, compared to the smooth strain M5, all three rough strains induced significantly higher LDH release from RAW264.7 cells at 24 h and 48 h p.i. (Figure 5a), indicating their strong macrophage-killing activity. Furthermore, a qPCR analysis of macrophage inflammatory responses revealed that infection with the rough strains for 24 h significantly upregulated the expression of pro-inflammatory cytokines IL−1β and IL−6 (Figure 5b,c), chemokines MCP1 and MIP1β (Figure 5d,e), and inducible nitric oxide synthase (iNOS) (Figure 5f) compared to the parental M5 strain. These results demonstrate that the three rough *Brucella* strains induce significant macrophage death and trigger robust inflammatory responses.

### 3.4. Rough Brucella Strains Exhibit Significantly Attenuated Virulence in Mice

**Figure 6:**
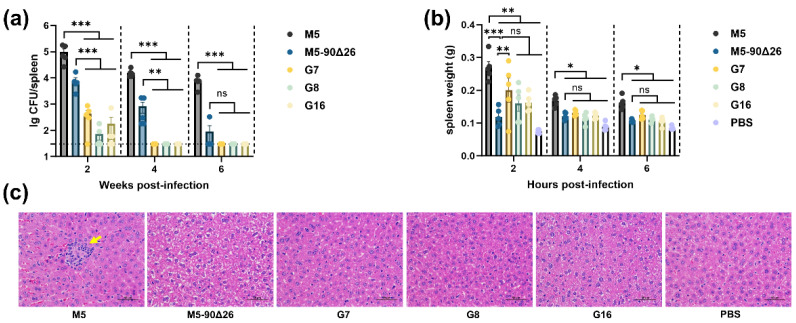
Virulence assessment of rough-type *Brucella* mutants. (**a**) Splenic bacterial load in mice infected with *Brucella* strains; (**b**) spleen weight of infected mice; (**c**) histopathological analysis of liver tissue from infected mice (magnification: 400×); yellow arrows indicate granulomas. Statistical significance was determined using two-way ANOVA (*, *p* < 0.05; **, *p* < 0.01; ***, *p* < 0.001, ns, not significant).

To evaluate the potential of three rough *Brucella* strains as vaccine candidates, we assessed their residual virulence in a mouse model. As shown in Figure 6a, at 2 and 4 weeks p.i., the bacterial loads in the spleens of mice infected with the vaccine strain M5–90Δ26 or the three rough strains (G7, G8, and G16) were significantly lower than those in mice infected with the parental strain M5. Moreover, the spleen bacterial loads in the rough strain-infected groups were markedly reduced compared to the M5–90Δ26 group. At 6 weeks p.i., both M5–90Δ26 and the rough strains resulted in significantly lower spleen bacterial loads than the M5 strain, with no significant difference observed between the vaccine strain and rough strains. Notably, no viable bacteria were isolated from the spleens of mice infected with the rough strains at 4 and 6 weeks p.i. (the dashed line in Figure 6a indicates the detection limit: 300 CFU/spleen). Spleen weight analysis revealed that at 2, 4, and 6 weeks p.i., mice infected with M5–90Δ26 or the rough strains exhibited significantly lower spleen weights than those infected with M5 (Figure 6b). Compared to M5–90Δ26, only the G7 strain caused a significantly higher spleen weight at 2 weeks p.i., while no significant differences were observed between the rough strains and M5–90Δ26 at 4 and 6 weeks p.i. (Figure 6b). To further assess pathogenicity, liver tissues were collected at 6 weeks p.i. for histopathological examination. Hematoxylin and eosin (H&E) staining demonstrated pronounced granuloma formation in the livers of M5-infected mice, whereas no granulomas were detected in mice infected with the rough strains (G7, G8, and G16) or M5–90Δ26 (Figure 6c). These results demonstrate that the three rough *Brucella* strains exhibit significantly attenuated virulence in mice, with residual virulence substantially lower than that of the vaccine strain M5–90Δ26.

### 3.5. Rough Brucella Strains Induce a Th1-Biased Cellular Immune Response in Vaccinated Mice

**Figure 7:**
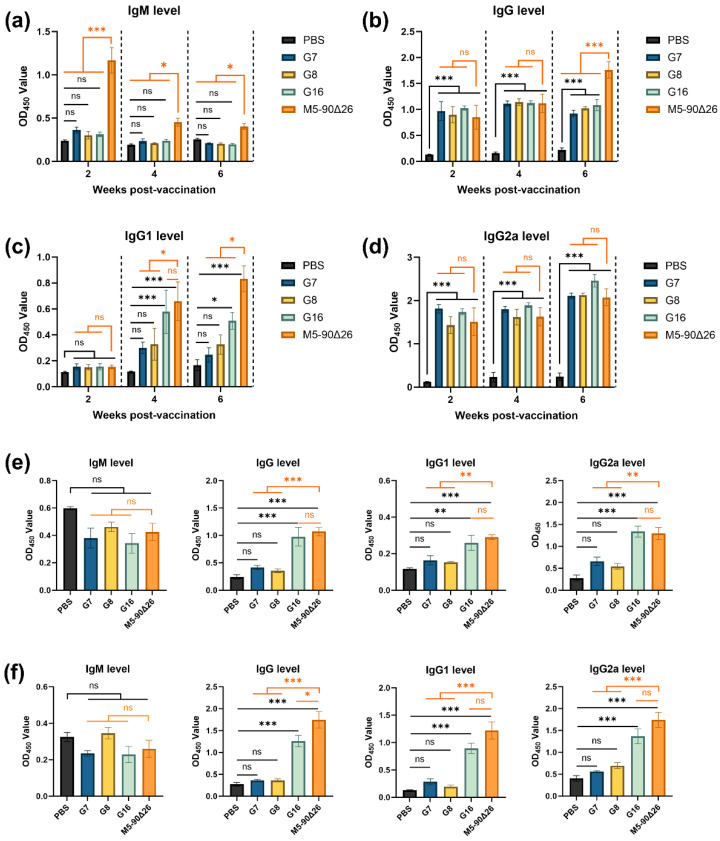
Antibody response analysis in vaccinated and challenged mice. (**a**) IgM levels in vaccinated mice; (**b**) IgG levels in vaccinated mice; (**c**) IgG1 levels in vaccinated mice; (**d**) IgG2a levels in vaccinated mice; (**e**) antibody levels and subclasses in mice challenged at 30 days post-vaccination; (**f**) antibody levels and subclasses in mice challenged at 45 days post-vaccination. Statistical significance was determined using one-way ANOVA or two-way ANOVA (*, *p* < 0.05; **, *p* < 0.01; ***, *p* < 0.001, ns, not significant).

To evaluate the immune response induced by rough *Brucella* strains, we measured IgM and IgG (including its subclasses) antibody levels in the serum of vaccinated and vaccinated-challenged mice using ELISA coated with total *Brucella* lysates. The results showed that serum IgM levels in mice vaccinated with rough strains were not significantly different from those in the PBS control group (Figure 7a). However, compared to the M5–90Δ26-vaccinated group, the rough *Brucella*-vaccinated mice exhibited significantly lower IgM levels. In contrast, both rough *Brucella* strains and M5–90Δ26 induced significantly higher levels of IgG and IgG2a than the PBS group at all post-vaccination (p.v.) time points (Figure 7b,d). Analysis of IgG1 levels revealed that only the semi-rough strain G16 showed a significantly higher IgG1 than the PBS group at 4 and 6 weeks p.v., although this was still lower than that in the M5–90Δ26 group. Meanwhile, G7 and G8 vaccination resulted in slightly elevated IgG1 levels, but the difference compared to the PBS group was not statistically significant (Figure 7c). Since IgG2a production is dependent on Th1-mediated cellular immunity, these findings suggest that the three rough *Brucella* strains primarily induce a Th1-biased immune response in mice.

To further assess immune memory levels, we collected serum from mice challenged at 30 and 45 days p.v. and measured IgM, IgG, and IgG subclass antibody levels via ELISA coated with total *Brucella* lysates. The results showed no significant differences in serum IgM levels among any groups (Figure 7e,f). In mice vaccinated with G7 or G8 and challenged with the parental M5 strain at 30 or 45 days p.v., IgG, IgG1, and IgG2a levels remained comparable to those in the PBS control group (Figure 7e,f). In contrast, G16- and M5–90Δ26-vaccinated mice exhibited significantly elevated IgG, IgG1, and IgG2a levels after M5 challenge compared to the PBS group. While G16-induced antibody levels were similar to those of M5–90Δ26 at 30 days p.v., the total IgG level in G16-vaccinated mice was notably lower than that in the M5–90Δ26 group upon challenge at 45 days p.v. These findings demonstrate that among the three rough *Brucella* strains, G16 induces a more robust immune memory response in mice.

### 3.6. Rough Brucella Strains G7 and G16 Provide Effective Immunoprotection in Mice

**Figure 8:**
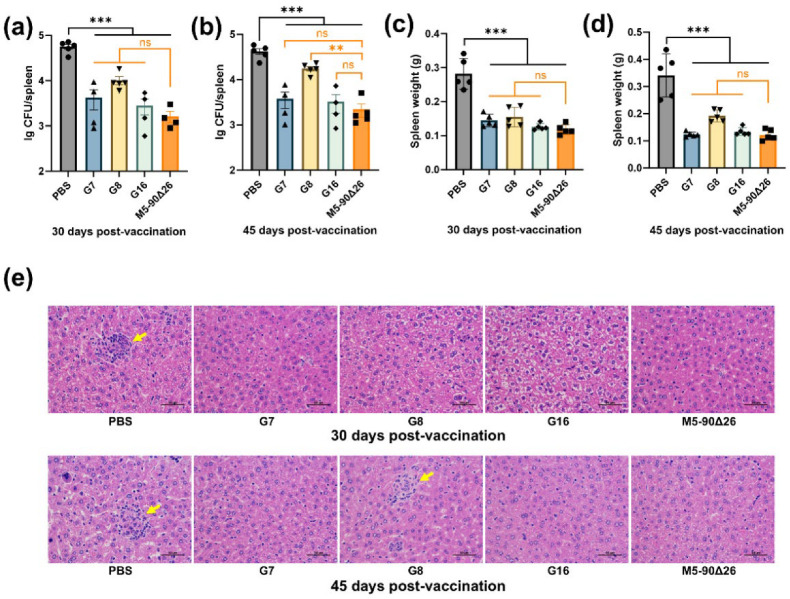
Protective immunity conferred by rough-type *Brucella* mutants. (**a**,**b**) Splenic bacterial load in mice at 30 or 45 days post-vaccination following challenge with parental strain M5; (**c**,**d**) spleen weight of mice challenged with M5 at 30 or 45 days post-vaccination; (**e**) hepatic histopathology in vaccinated mice post-challenge (magnification: 400×); yellow arrows indicate granulomas. Statistical significance was determined using one-way ANOVA (**, *p* < 0.01; ***, *p* < 0.001, ns, not significant).

To evaluate the immunoprotective efficacy of three rough *Brucella* vaccine strains in mice, animals were challenged intraperitoneally with the M5 strain at 30 and 45 days p.v. The results demonstrated that mice vaccinated with any of the three rough *Brucella* strains exhibited significantly reduced bacterial loads in the spleen compared to the PBS control group (Figure 8a,b). No significant difference in splenic bacterial burden was observed between the G7 or G16 vaccination groups and the M5–90Δ26 group (J.Qa and 8b). However, the G8-vaccinated group showed a significantly higher bacterial load at 45 days p.v. after M5 challenge (Figure 8b). Spleen weight analysis revealed that all three rough *Brucella* strains significantly reduced splenomegaly compared to the PBS group, with no significant difference from the M5–90Δ26 group (Figure 8c,d). Histopathological examination showed no notable liver lesions in either rough *Brucella*-vaccinated or M5–90Δ26-vaccinated mice at 30 days p.v. after challenge, whereas PBS-control mice developed distinct granulomas (Figure 8e). At 45 days p.v. after challenge, the G7 and G16 groups maintained a normal liver histopathology comparable to the M5–90Δ26 group, while sporadic granulomas appeared in some G8-vaccinated mice, and severe granulomatous inflammation persisted in PBS controls (Figure 8e). These findings collectively indicate that rough *Brucella* strains G7 and G16 confer robust immunoprotection in murine models; however, strain G8 had weaker immune protection against mice than other strains.

### 3.7. Rough Brucella Strains Can Be Differentially Diagnosed Using the LPS-ELISA Method

**Figure 9:**
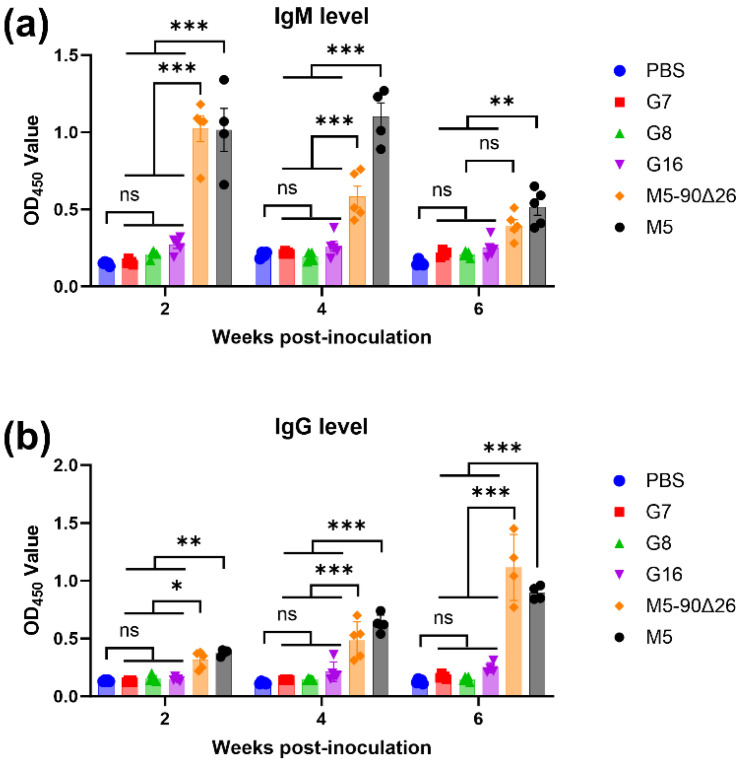
Differential diagnosis of *Brucella* infection and vaccinated mice using LPS-coated ELISA. (**a**) IgM levels detected by LPS-ELISA in inoculated mice; (**b**) IgG levels detected by LPS-ELISA in inoculated mice. Statistical significance was determined using two-way ANOVA (*, *p* < 0.05; **, *p* < 0.01; ***, *p* < 0.001, ns, not significant).

The challenge in differential diagnosis is one of the major limitations for the application of *Brucella* vaccines [20]. To evaluate whether the rough *Brucella* strains obtained in this study could be distinguished from smooth strains, we established an LPS-ELISA method for smooth *Brucella* and measured IgM and IgG levels in serum samples from mice inoculated with PBS, three rough *Brucella* strains, M5–90Δ26, or M5. The results showed that at 2, 4, and 6 weeks post-inoculation, IgM levels detected by LPS-ELISA in mice inoculated with the three rough *Brucella* strains showed no significant difference compared to the PBS control group but were significantly lower than those in mice inoculated with the smooth strains M5–90Δ26 and M5 (Figure 9a). Similarly, IgG levels in the rough *Brucella*-inoculated groups exhibited no significant difference from the PBS group and were significantly lower than those in the M5–90Δ26 and M5 groups (Figure 9b). Furthermore, experimental data revealed that at 2 and 4 weeks post-inoculation, the difference in serum IgM levels between rough and smooth strain-inoculated mice was more pronounced than the difference in IgG levels. In contrast, at 6 weeks post-inoculation, the difference in IgG levels became more significant than that of IgM. These results demonstrate that the simultaneous detection of IgM and IgG levels via LPS-ELISA can effectively differentiate mice inoculated with rough *Brucella* strains from those inoculated with smooth strains. Thus, rough *Brucella* strains represent a promising candidate for DIVA vaccines.

## 4. Discussion

### 4.1. Three Rough-Type Brucella Strains with Spontaneous O-Antigen Loss Exhibit Attenuation and Vaccine Potential

The GntR family of transcriptional regulators represents a crucial class of regulatory elements in *Brucella*. To date, more than 20 GntR regulators have been identified in *Brucella* [11], with several reported to attenuate bacterial virulence upon deletion. In this study, we found that the deletion of *gntR7*, *gntR8*, and *gntR16* significantly reduced the virulence of *B. melitensis* strain M5. However, genetic complementation (restoring gene expression at the native locus) failed to restore virulence in any of the three mutants. Further investigation revealed that all three mutants exhibited loss of LPS O-antigen. This finding is not surprising, as LPS is a critical virulence factor in *Brucella*, composed of lipid A, core oligosaccharide and O-antigen [21]. O-antigen is classical structure that confers peculiar characteristics to *Brucella* LPS tics, and instability in O-antigen structure often leads to a transition from a smooth to a rough phenotype, accompanied by a marked decline in virulence [22]. Rough *Brucella* strains typically display higher OD_600_ values and increased susceptibility to polymyxin B [17,18], traits also observed in the G7, G8, and G16 mutants in this study. Interestingly, compared with the parental strain M5, the G16 mutant only retained a partial O-antigen structure as detected by Western Blotting, yet still exhibited similar biological characteristics to rough strains. Notably, a detailed structural characterization of the G16 mutant’s LPS-like core oligosaccharide and lipid A was not performed in this study, limiting our understanding of its semi-rough phenotype and immunological implications. Nevertheless, given that rough *Brucella* strains are attenuated and do not induce O-antigen-specific antibodies in vaccinated animals, they serve as ideal vaccine candidates that do not interfere with clinical diagnostics—as exemplified by the commercially available rough *B. abortus* vaccine strain RB51 [23]. Currently, no rough *B. melitensis* vaccine is available, prompting us to evaluate the potential of these three rough mutants as candidate vaccines.

### 4.2. Attenuated Rough Brucella Strains G7/G8/G16 Show Hyper-Adhesion, Cytotoxicity, and Immune Activation

Phenotypic virulence analysis showed that G7, G8, and G16 exhibited enhanced adhesion and invasion capabilities toward macrophages but significantly impaired intracellular survival. The increased adhesion and invasion may be attributed to differences in macrophage receptor interactions between rough and smooth *Brucella* strains. For instance, while the macrophage uptake of smooth *Brucella* depends on PI3-kinase and Toll-like receptor 4 (TLR4), rough strains are internalized independently of these factors [24]. Additionally, rough *Brucella* fails to evade lysosomal degradation post-invasion, leading to reduced intracellular survival [25]—a phenotype consistent with the observed attenuation of G7, G8, and G16. Notably, artificially derived rough *Brucella* strains often exhibit macrophage cytotoxicity [15,19], and as expected, G7, G8, and G16 also demonstrated this trait. Studies suggest that rough *Brucella* strains exhibit elevated expression and secretion activity of the Type IV secretion system (T4SS), which mediates cytotoxicity [15]. The three rough mutants in this study may employ a similar mechanism, though further validation of T4SS expression is required. Furthermore, cytokine and chemokine profiling revealed that rough *Brucella* infection triggers stronger immune cell activation, potentially accelerating bacterial clearance. This heightened immune response may enhance host resistance, as evidenced by the near-undetectable levels of G7, G8, and G16 in mouse spleens four weeks p.i. and the absence of granuloma formation in liver histopathology—consistent with the attenuated virulence of rough *Brucella* [17,18]. Compared to the commercial vaccine strain M5–90Δ26, G7, G8, and G16 displayed lower residual virulence, suggesting superior potential as live-attenuated vaccines.

### 4.3. G16, a Semi-Rough Brucella Mutant, Induces Robust Th1 Immunity and Memory Response, Outperforming Other Vaccine Candidates

Protective immunity against *Brucella* primarily relies on Th1-mediated cellular immune responses [26]. In mice, Th1 responses drive IgG2a production, while Th2 responses induce IgG1 [27]. Here, all three rough vaccine candidates and M5–90Δ26 elicited high IgG2a levels, indicating Th1-biased immunity—a pattern consistent with the rough *Brucella* RB51 vaccine [28]. However, post-challenge, only G16 induced IgG2a levels comparable to M5–90Δ26, suggesting superior Th1 memory responses compared to G7 and G8. The underlying mechanism remains unclear, warranting further investigation. Additionally, only M5–90Δ26 triggered significant IgM production, particularly in early immunization phases. This aligns with reports that *Brucella* infection initially induces IgM, later shifting to IgG [29]. Intriguingly, rough *Brucella* strains, including RB51, do not elicit detectable IgM, implying that IgM responses primarily target O-antigen [30].

### 4.4. G16’s Semi-Rough Phenotype Confers Robust Protection, Outperforming Fully Rough Brucella Vaccine Strains

Protective efficacy is a key metric for *Brucella* vaccine candidates. Here, G7 and G16 conferred protection comparable to M5–90Δ26, supported by histopathological findings. Despite similar antibody profiles, G7 and G8 exhibited divergent protective outcomes, possibly due to differences in cellular immunity undetectable by IgG subclass analysis. Future studies employing flow cytometry to assess CD4^+^/CD8^+^ T cell subsets, cytokine profiles, and memory T cell activation may clarify this discrepancy. G16’s robust protection, mirroring its antibody response, may stem from its partial O-antigen retention. Vemulapalli et al. reported that complementing RB51 with *whoA* to restore O-antigen synthesis enhanced vaccine efficacy [30], echoing our findings with G16. This suggests that semi-rough *Brucella* vaccines may offer superior protection over fully rough strains.

### 4.5. All Three Rough Brucella Vaccine Candidates Enable Reliable DIVA Discrimination via LPS-ELISA

A key advantage of rough vaccines is their compatibility with DIVA strategies. The rough RB51 strain remains the only universally accepted DIVA vaccine for *Brucella* [31]. Here, LPS-ELISA confirmed that rough G7 and G8 elicited low levels of IgM or IgG, as expected. Surprisingly, G16—despite its partial O-antigen retention—also failed to induce detectable antibodies, possibly due to insufficient O-antigen immunogenicity or rapid in vivo attenuation. Notably, IgM and IgG monitoring effectively distinguished rough vaccine immunization from wild-type infection at early and late stages, respectively. In conclusion, LPS-ELISA-based IgM/IgG detection reliably differentiates rough vaccine immunization from natural infection.

### 4.6. Translational Challenges and Safety Considerations for Rough Brucella DIVA Vaccines

While the rough-type *Brucella* strains G7, G8, and G16 demonstrate potential as DIVA-compatible vaccine candidates, several critical considerations must be addressed before clinical translation. First, field implementation may encounter challenges, including (1) serological cross-reactivity in regions with mixed *Brucella* species circulation, (2) heterogeneous immune responses in endemic livestock populations, and (3) the limitations of diagnostic infrastructure in resource-limited settings. Second, safety concerns common to live attenuated vaccines require thorough evaluation—as evidenced by the historical experience with rough vaccine RB51, which shows residual virulence, contraindication in pregnant animals, and diagnostic interference through false-positive serological results [32]. These issues underscore the need for comprehensive safety profiling, including (i) an assessment of vertical transmission risk, (ii) an evaluation of environmental persistence, and (iii) validation of DIVA specificity in target species. Furthermore, genetic heterogeneity in the mutant strains (G7, G8, G16) due to potential off-target mutations or mixed populations may affect vaccine consistency and safety. Additionally, this study’s limitations—particularly the use of murine models that may not fully recapitulate natural host–pathogen interactions, and controlled laboratory conditions that differ from field environments—necessitate further validation in target livestock species under operational conditions. Rigorous large-scale efficacy trials and risk–benefit analyses will be essential before clinical deployment of these vaccine candidates.

## Figures and Tables

**Table 1:** Bacterial strains and plasmids used in this study.

Names	Description	Source
**Bacterial strains**		
M5	*B. melitensis* parental strain; low virulence; smooth phenotype	CVCC
M5–90Δ26	*B. melitensis* vaccine strain; smooth phenotype	CVCC
G7	*gntR7* deletion mutant derived from M5; rough phenotype	This study
G8	*gntR8* deletion mutant derived from M5; rough phenotype	This study
G16	*gntR16* deletion mutant derived from M5; semi-rough phenotype	This study
*E. coli* DH5α	F^−^, φ80d*lacZ* ΔM15, Δ *(lacZYA-argF)* U169, *recA1*, *endA1*, *hsdR17 (rk^−^, mk^+^)*, *phoA*, *supE44*, *thi−1*, *gyrA96*, *relA1*, *λ^−^*	TIANGEN
**Plasmids**		
pKB	pUC19-derived suicide plasmid containing *sacB* gene; Kan^R^;	[12]
pKB-ΔgntR7	pKB containing the upstream and downstream fragments of the *gntR7* gene	This study
pKB-ΔgntR8	pKB containing the upstream and downstream fragments of the *gntR8* gene	This study
pKB-ΔgntR16	pKB containing the upstream and downstream fragments of the *gntR16* gene	This study

**Table 2:** Primers used in this study.

Primers	Sequence (5′-3′)	Function
GntR7-UF	GGTACCCGGGGATCCGCGGCATTGGGGCTGAAGCG	PCR amplification of the homologous fragments of the *gntR7* gene
GntR7-UR	TGACGGGTTTTGCTAGTCATTCCTCCTCGACTTCA	
GntR7-DF	TCGAGGAGGAATGACTAGCAAAACCCGTCAGGCCG	
GntR7-DR	TGCCTGCAGGTCGACAGAGCGCGGTCAAGGTGGCG	
inG7-F	GCCTGAAGAAATTGCTCGAC	PCR identification of the G7 strain
inG7-R	GCGCGTTTGATCTGCTTCAG	
outG7-F	GTCTGGTTGACTTGTTTGAC	
outG7-R	TGGCTGATCGGGCTTATCTC	
GntR8-UF	GGTACCCGGGGATCCAGGCCGCAAGCTCCGCCTCG	PCR amplification of the homologous fragments of the *gntR8* gene
GntR8-UR	AGTACCGAAAATGTTGCTGCTTCTTGCTTTGTGAC	
GntR8-DF	AAAGCAAGAAGCAGCAACATTTTCGGTACTGGCCA	
GntR8-DR	TGCCTGCAGGTCGACCACTGGAATCGCGTCAGGTG	
inG8-F	GTTAAAGCGCACGAAACATC	PCR identification of the G8 strain
inG8-R	GCTTCGAGCCTCTCGTAATC	
outG8-F	CGTGTGGAATGTCTATCGAT	
outG8-R	GATGTGCGTGAAGATCTGCT	
GntR16-UF	GGTACCCGGGGATCCACCCAGCCCCTGAATAATGC	PCR amplification of the homologous fragments of the *gntR16* gene
GntR16-UR	AAAGTGGCACGAAGGCAATAGGAAAGCCTCCCAAC	
GntR16-DF	GAGGCTTTCCTATTGCCTTCGTGCCACTTTTACGC	
GntR16-DR	TGCCTGCAGGTCGACCAAGGCTGCGGCCCAGAATC	
inG16-F	GATTTATGCGGGCGATTATG	PCR identification of the G16 strain
inG16-R	ATCACCGCCATGTGAAAATC	
outG16-F	GTAGATATTCCGGTCGTTTT	
outG16-R	CCCCATCTTATTTTCTTGCG	
RT-IL1B-F	TGGACCTTCCAGGATGAGGACA	qPCR for the IL−1β gene
RT-IL1B-R	GTTCATCTCGGAGCCTGTAGTG	
RT-IL6-F	TACCACTTCACAAGTCGGAGGC	qPCR for the IL−6 gene
RT-IL6-R	CTGCAAGTGCATCATCGTTGTTC	
RT-MCP1-F	GCTACAAGAGGATCACCAGCAG	qPCR for the MCP1 gene
RT-MCP1-R	GTCTGGACCCATTCCTTCTTGG	
RT-MIP1B-F	ACCCTCCCACTTCCTGCTGTTT	qPCR for the MIP1β gene
RT-MIP1B-R	CTGTCTGCCTCTTTTGGTCAGG	
RT-iNOS-F	GAGACAGGGAAGTCTGAAGCAC	qPCR for the iNOS gene
RT-iNOS-R	CCAGCAGTAGTTGCTCCTCTTC	
RT-ActB-F	CATTGCTGACAGGATGCAGAAGG	qPCR for the β-actin gene
RT-ActB-R	TGCTGGAAGGTGGACAGTGAGG	

## Data Availability

The original contributions presented in this study are included in the article. Further inquiries can be directed to the corresponding author.

## Abbreviations

The following abbreviations are used in this manuscript:

DIVA	Differentiating Infected from Vaccinated Animals
LDH	Lactate dehydrogenase
qPCR	Quantitative polymerase chain reaction
ELISA	Enzyme-linked immunosorbent assay
LPS	Lipopolysaccharide
CFU	Colony-forming unit
